# Adult Mouse Subventricular Zone Stem and Progenitor Cells Are Sessile and Epidermal Growth Factor Receptor Negatively Regulates Neuroblast Migration

**DOI:** 10.1371/journal.pone.0008122

**Published:** 2009-12-02

**Authors:** Yongsoo Kim, Isabelle Comte, Gabor Szabo, Philip Hockberger, Francis G. Szele

**Affiliations:** 1 Interdepartmental Neuroscience Program, Northwestern University, Chicago, Illinois, United States of America; 2 Department of Gene Technology and Developmental Neurobiology, Institute of Experimental Medicine, Budapest, Hungary; 3 Department of Physiology, Feinberg School of Medicine, Northwestern University, Chicago, Illinois, United States of America; 4 Department of Physiology, Anatomy and Genetics, University of Oxford, Oxford, United Kingdom; Institut de la Vision, France

## Abstract

**Background:**

The adult subventricular zone (SVZ) contains stem and progenitor cells that generate neuroblasts throughout life. Although it is well accepted that SVZ neuroblasts are migratory, recent evidence suggests their progenitor cells may also exhibit motility. Since stem and progenitor cells are proliferative and multipotential, if they were also able to move would have important implications for SVZ neurogenesis and its potential for repair.

**Methodology/Principal Findings:**

We studied whether SVZ stem and/or progenitor cells are motile in transgenic GFP+ slices with two photon time lapse microscopy and *post hoc* immunohistochemistry. We found that stem and progenitor cells; mGFAP-GFP+ cells, bright nestin-GFP+ cells and Mash1+ cells were stationary in the SVZ and rostral migratory stream (RMS). In our search for motile progenitor cells, we uncovered a population of motile βIII-tubulin+ neuroblasts that expressed low levels of epidermal growth factor receptor (EGFr). This was intriguing since EGFr drives proliferation in the SVZ and affects migration in other systems. Thus we examined the potential role of EGFr in modulating SVZ migration. Interestingly, EGFr^low^ neuroblasts moved slower and in more tortuous patterns than EGFr-negative neuroblasts. We next questioned whether EGFr stimulation affects SVZ cell migration by imaging Gad65-GFP+ neuroblasts in the presence of transforming growth factor alpha (TGF-α), an EGFr-selective agonist. Indeed, acute exposure to TGF-α decreased the percentage of motile cells by approximately 40%.

**Conclusions/Significance:**

In summary, the present study directly shows that SVZ stem and progenitor cells are static, that EGFr is retained on some neuroblasts, and that EGFr stimulation negatively regulates migration. This result suggests an additional role for EGFr signaling in the SVZ.

## Introduction

The adult subventricular zone (SVZ) is one of two largest neurogenic areas of the adult brain [1]. The current model of the adult SVZ delineates three neurogenic cell types: glial fibrillary acidic protein (GFAP+) stem cells, epidermal growth factor receptor (EGFr+) transit-amplifying progenitor cells, and doublecortin (Dcx+) neuroblasts [2], [3]. Stem cells divide slowly and generate transit-amplifying progenitor cells which divide rapidly to produce neuroblasts [4], [5]. Using ^3^H-thymidine and histological analyses, Altman showed that neuroblasts migrate in the rostral migratory stream (RMS), a densely packed corridor of cells moving from the SVZ to the olfactory bulbs [6]. These and many other studies of migration were static experiments that determined the final position of labeled cells or used cell morphology to assess migration. There are several shortcomings with these approaches. First, one can never be certain of the trajectory taken by a migrating cell between its point of origin and final position. In addition, local motility would not be detected with dye, thymidine analogue or retroviral labeling and static histological approaches. Indeed, two-photon time lapse studies revealed that in addition to long-distance migration, one third of motile SVZ cells move in local exploratory patterns [7]. Finally, migratory morphology is not always correlated with motility, motile cells can change shape dramatically [7]. Thus local motility of stem and progenitor cells, or even rare long-distance motility may have been missed with previous approaches. If pluripotential stem cells or rapidly dividing progenitor cells migrate in the SVZ, they may also migrate to injuries and be more reparative than SVZ neuroblasts, which are fate-restricted.

Studies have emerged recently which suggest that transit-amplifying progenitor cells in the SVZ may be motile. A population of 2′,3′-cyclic nucleotide 3′-phosphodiesterase-enhanced green fluorescent protein/NG2+ cells were identified as migratory transit-amplifying progenitor cells [8], [9]. Other studies found motile nestin-GFP+ cells that did not express Dcx, suggesting stem cells or progenitor SVZ cells could be motile [7] since Dcx is thought to be expressed only in SVZ neuroblasts [10], [11]. In addition, astrocytes and progenitors can be motile in a variety of developmental systems and after injury [12], [13]. Finally, cells with SVZ progenitor cell characteristics emigrate to the striatum after injury and growth factor infusion [14].

Epidermal growth factor receptor (EGFr) is thought to be expressed on stem cells and transit-amplifying progenitor cells in the SVZ, but to be absent from neuroblasts [2], [15]. EGFr stimulation serves largely to drive proliferation in the SVZ: EGF infusion into the lateral ventricle increases proliferation [16] and TGF-α null mice have reduced SVZ cell proliferation [17]. EGF is also necessary for driving proliferation and self-renewal in the *in vitro* neurosphere assay, confirming its central role in SVZ proliferation [15], [18], [19]. Interestingly, EGF exposure reverts the transit-amplifying population to a more stem cell-like state, suggesting EGFr regulates the balance of SVZ cell subtypes [15]. Other data show EGFr signaling plays a role in modulating migration in the forebrain. Interestingly overexpression of EGFr confers migratory properties on SVZ and other telencephalic cells [20]–[22]. Infusing EGF into the lateral ventricle not only modulates proliferation, but also results in SVZ cells migrating from their normal route and into the adjacent striatum and septum [15], [16]. Finally, nigrostriatal denervation combined with TGF-α infusion into the striatum induced emigration of SVZ cells [23], [24]. Thus we were interested in the possibility that EGFr modulates SVZ cell migration.

In this study we first studied transit-amplifying progenitor and stem cell motility in the adult SVZ. We looked for GFP labeled SVZ cell subtype motility with two photon microscopy of acute slices and followed it with *post hoc* immunohistochemistry to further examine phenotypes. We show here that stem cells and transit-amplifying progenitor cells are stationary whereas neuroblasts are motile. We also show that some neuroblasts retained EGFr expression and its stimulation is negatively correlated with SVZ cell motility.

## Methods

### Animal

**Figure 1:**
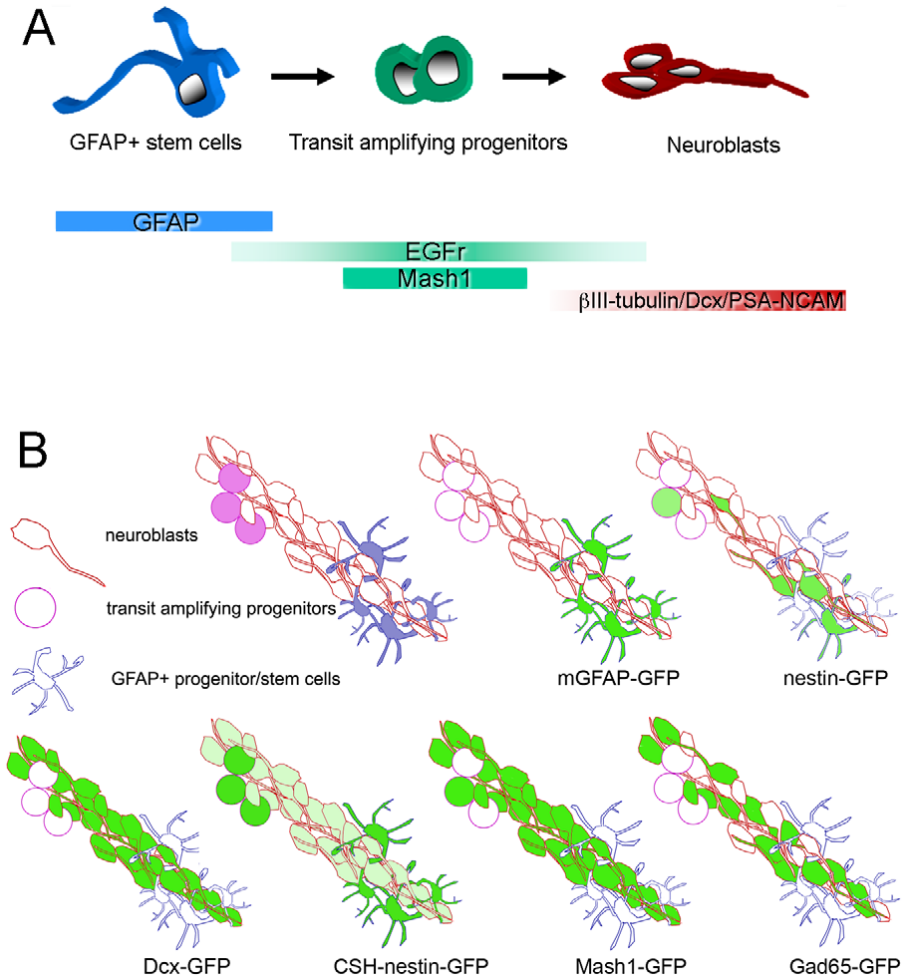
Subventricular zone cell types selectively labeled with GFP. A: Stem cells in the SVZ express GFAP and give rise to EGFr+ and Mash1+ transit-amplifying progenitors. These, in turn generate migratory neuroblasts that express βIII-tubulin, Dcx, and PSA-NCAM. Note that the expression and loss of some markers, such as EGFr is gradual. B: Model of a typical SVZ neuroblast chain (red) with cluster of transit-amplifying progenitors (purple) and GFAP+ astrocytes (blue) surrounding it. For the sake of clarity only a few progenitors (top of chain) and GFAP+ cells (bottom of chain) are shown. mGFAP-GFP mice only label GFAP+ cells. Nestin-GFP labels a subset of all three cell types and Dcx-GFP labels all and only neuroblasts. The CSH-nestin-GFP line labels stem cells and transit-amplifying progenitors GFP^bright^ and neuroblasts GFP^dim^. Unexpectedly, the Mash1-GFP mouse labels not only the transit-amplifying progenitors but also neuroblasts. The Gad65-GFP mouse labels a subset of neuroblasts. Adapted from [7].

Breeder mice were obtained from Vijay Sarthy (Northwestern U., mGFAP-GFP), Grigori Enikolopov (Cold Spring Harbor Lab, “CSH-nestin-GFP”), Anjen Chenn (Northwestern U., “Nestin-GFP”), the NIH Gensat Project (Rockefeller U., Mash1-GFP and Dcx-GFP) [25], and Gábor Szabo (Institute of Experimental Medicine, Gad65-GFP). Details of the mGFAP-GFP mouse line [26], the CSH-Nestin-GFP line [27], the Nestin-GFP line and the Dcx-GFP line [7], and the Gad65-GFP line [28] are published elsewhere and summarized in Fig. 1B. The mice used were 1–2 months old. All animals were handled in strict accordance with good animal practice as defined by the UK Animals (Scientific Procedures) 1986 Act, UK Home Office and NIH guidelines. All animal work was approved by the UK Home Office, License #30/2496, and the University of Oxford Department of Physiology, Anatomy and Genetics Departmental Ethical Review Committee.

### Slice Preparation and Two Photon Time Lapse Imaging

Details of our two photon (2P) imaging are as previously described [7]. Briefly, mice were anesthetized with isoflurane (0.25 ml/L for 1 min), immersed in ice for 5 min, decapitated and the brain quickly removed, placed in ice-cold artificial cerebrospinal fluid (aCSF; 125 mM NaCl, 2.5 mM KCl, 2 mM, CaCl^2^, 1 mM MgCl^2^, 26 mM NaHCO^3^, 1.25 mM H_2_PO^4^, and 25 mM glucose at pH 7.4). The brain was bisected and trimmed for mounting. Hemispheres were mounted on a platform, submerged in ice-cold aCSF and 300 µm sagittal slices cut on a Vibratome (Campden Instruments Ltd.). Slices were collected sequentially in a chamber filled with oxygenated aCSF, incubated at 35°C for 40 min, and returned to RT (about 1 hr) before imaging. For cell imaging, each section was examined shortly under low power epifluorescence to find the optimal slice and field. A two photon laser (Mira 900, Coherent) was used to acquire 51 optical sections separated by 1 µm, every 3 min. Oxygenated aCSF was constantly perfused during the imaging at 0.5–1.0 ml/min. For TGF-α experiments, 10 ng/ml TGF-α was dissolved in oxygenated aCSF and either TGF-α, or aCSF as a control, constantly perfused during imaging at 0.2–0.5 ml/min.

### Image Processing and Quantification

Two photon images were acquired as a stack of TIFF images using Fluoview software (Olympus) and data processed with Volocity software (Improvision). Briefly, Fluoview files were imported into Volocity, decompressed and processed with autocontrast, fine median filter, and auto level functions to obtain optimal image quality. Each stack of processed data was compressed and exported into Quicktime (Apple). For quantifying cell speed and motility, 3D coordinates of cells in each frame were recorded and calculated using Volocity. If the net distance of displacement divided by the total migration distance was less than 0.4, between 0.4 and 0.6, or more than 0.6, cells were classified as exploratory, intermediate, or migratory, respectively. Cell speed was plotted by dividing total migration distance/time. For percentage migratory cell analysis in the TGF-α experiments, the first hour of pre-treatment and the last hour of drug treatment were quantified. Cells in the first frame of each movie were numbered and their motility subsequently followed in 2P movies. Only cells moving more than two cell diameters were considered motile.

### Immunohistochemistry and Confocal Microscopy

For *post hoc* immunohistochemistry, immediately after the last 2P frame was taken slices were fixed with 4% paraformaldehyde for 1 hr and transferred to cryoprotectant at 4°C. Slices were washed with PBS 3 times for 10 min, 50 mM glycine for 15 min, PBS 3 times for 10 min, PBS+ (10% Donkey Serum/0.7% Triton X-100 in PBS) for one hour, and incubated in primary antibodies [sheep anti-EGFr (1∶50, Upstate), mouse anti-Mash1 (1∶200, BDscience), rabbit anti-EGFr (1∶200, Santa Cruz, sc-03), rabbit anti-Phosphohistone 3 (1∶500, Millipore), rabbit anti-caspase-3 (1∶200, Cell Signalling Technology), or goat anti-Dcx (1∶100, Santa Cruz) in PBS+] at 4°C for two days. Then, slices were washed with PBS 3 times 10 min, Cy3 donkey anti-sheep, donkey anti-rabbit or donkey anti-mouse (all 1∶500, Jackson Immunoresearch) or Cy5 donkey anti-goat (1∶500, Jackson Immunoresearch) in PBS+ for 1.5 hr, PBS 3 times 10 min, DAPI (40 mg/mL DAPI stock solution 1∶1000 in PBS) for 10 min, PB 3 times 10 min, air dried and coverslipped with Fluorsave (Calbiochem). Immunostained slices were examined with confocal microscopy (Zeiss LSM510 or LSM710). The 2P imaged area was found by comparing the last frame of 2P imaging with the confocal image, and matching major blood vessels and static GFP+ cells. Once matched GFP+ cells were identified, they were numbered and each was examined for motility patterns and speed in the time lapse movie and for marker expression with *post hoc* immunohistochemistry.

For standard immunohistochemistry, mice were perfused under deep anaesthesia with 4% paraformaldehyde. 30 µm sections were collected on a sliding microtome (Leica) and the same procedure was used as above except primary antibody incubation was overnight, 0.1% triton X-100 in PBS, and 1 hr secondary incubation. Primary antibodies; goat anti-Dcx (1∶200, Santa Cruz), mouse anti-βIII-tubulin (1∶500, Covance), sheep anti-EGFr (1∶50, Upstate), or rabbit anti-EGFr (1∶200, Santa Cruz, sc-03), or mouse anti-Mash1 (1∶200, BDscience). For triple neuroblast staining; rabbit anti-Tuj1 (1∶2000, Covance), mouse anti-PSA-NCAM (1∶500, Chemicon), goat anti-Dcx (1∶100, Santa Cruz). Secondary antibodies; Alexa488 anti-mouse (1∶500, Invitrogen), Cy3 anti-goat or sheep, Cy5 anti-mouse (1∶500, Jackson Immunoresearch). All immunohistochemistry analysis was done on N≥3 mice.

### *In Vivo* Dye Injection and Quantification

Cell Tracker Orange CMTMR (CTO, Molecular Probes) was reconstituted in DMSO to a final concentration of 10 mM [29]. Briefly, each animal was deeply anesthetized with 150 mg/kg ketamine, 10 mg/kg xylazine, i.p., placed in a stereotaxic apparatus (Stoelting, Wood Dale, IL). A burr-hole was made for Hamilton syringe insertion and 1 µl of CTO was injected over 1 min into the lateral ventricle (LV) (sterotactic coordinates: Bregma, A/P: +0.26, L/M: +0.75, D/V:−2.5) of 2 month old Dcx-GFP mice. Animals were placed on a warm pad and monitored until recovered. 3 days after injection, mice were perfused as above. Injection into the LV was confirmed in each mouse with CTO diffusion all the way to the contralateral LV.

### Statistics

Statistical differences were determined by Student's T-test, and distributions represented by SEM (standard error of mean).

## Results

### SVZ Stem Cells Were Stationary

**Figure 2:**
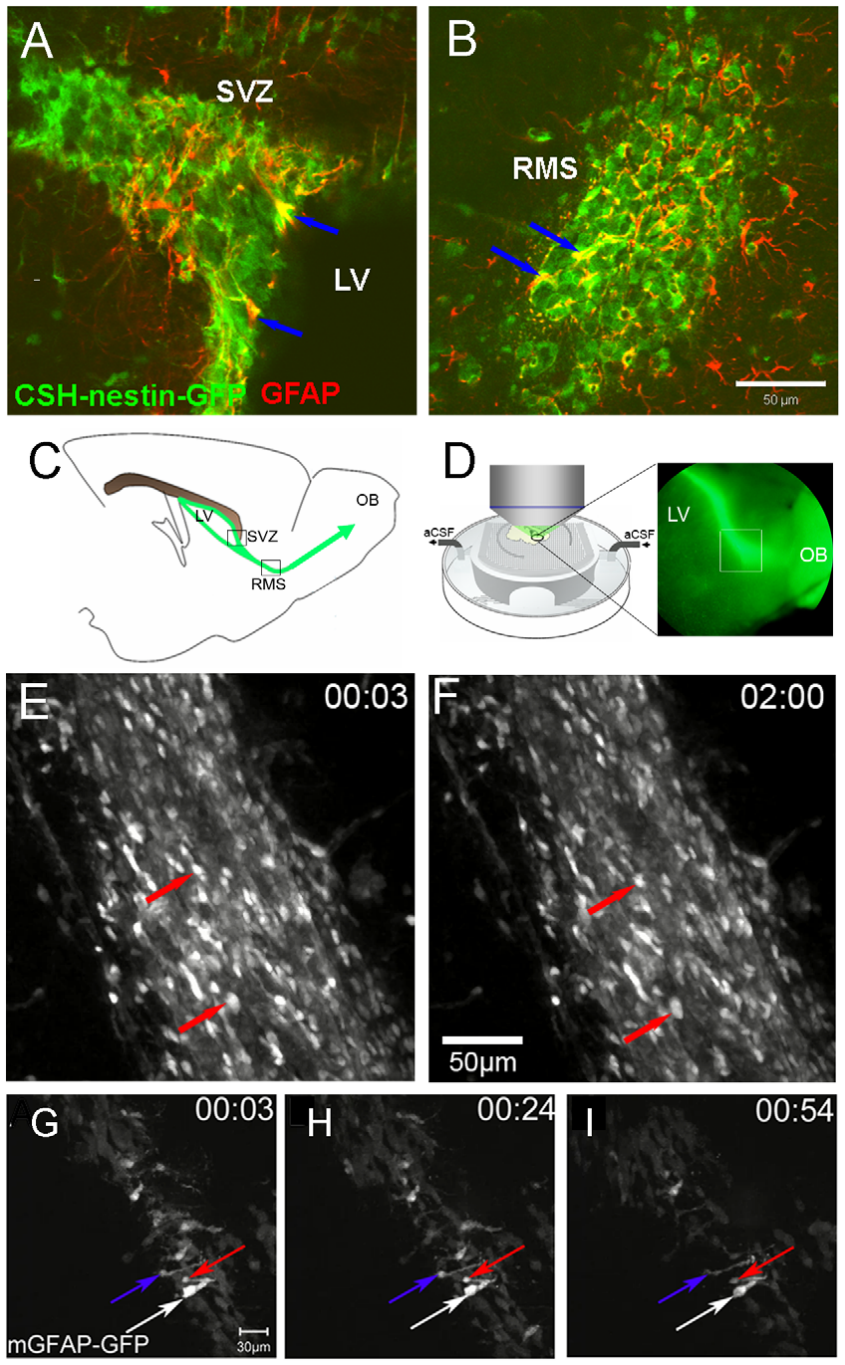
Stem and progenitor cells are stationary in the SVZ. A–B: CSH-nestin-GFP showed bright GFP+ cells colocalized with GFAP immunohistochemistry (blue arrows) in the SVZ (A) and the RMS (B), (coronal sections). Scale bar = 50 µm. C: Location of two photon imaging in the SVZ and RMS of sagittal slices. LV = lateral ventricle, OB = olfactory bulb. D: Schematic of two photon imaging and a 5X image of the RMS in a CSH-nestin-GFP mouse. E-F: Bright CSH-nestin-GFP+ cells (ex. red arrows) showed no local movement in the RMS in two photon time lapse imaging. Time stamp is in hr:min in all figures and movies. G–I: All mGFAP-GFP+ cells in the movie were stationary during imaging. Examples of individual cells are indicated with arrows. Scale bar = 30 µm.

We previously characterized and examined motility with two photon time lapse microscopy in a nestin-GFP mouse that primarily labels SVZ neuroblasts (Fig. 1B), [7]. We began this study using a different nestin-GFP mouse (“CSH-nestin-GFP”), in which GFP^bright^ cells are neurosphere-forming SVZ stem and progenitor cells and GFP^dim^ cells are neuroblasts (Fig. 1B), [27]. Stem cells in the SVZ express GFAP [4], [5] (Fig. 1A), and we found that many GFP^bright^ cells in the SVZ and the RMS colocalized with GFAP immunostaining (Fig. 2A,B). We utilized two photon microscopy to determine if CSH-nestin-GFP^bright^ cells are motile (Fig. 2C,D). GFP^bright^ cells in the SVZ and RMS did not move during the two hour recording period (N = 4 mice, 198 cells in the SVZ and 586 cells in the RMS) (Fig. 2E,F; Movie S1) while GFP^dim^ neuroblasts were actively moving, similar to our previous results with nestin-GFP mice [7]. We next used a mGFAP-GFP line to further study potential stem cell motility. Every mGFAP-GFP+ cell examined in the SVZ and RMS was stationary (N = 3 mice, 65 cells analyzed, 1 hr time lapses) (Fig. 2G–I). These data suggest that GFAP+ astrocyte-like stem cells are static in the adult SVZ.

### Transit-Amplifying Progenitor Cells Were Also Non-Motile

**Figure 3:**
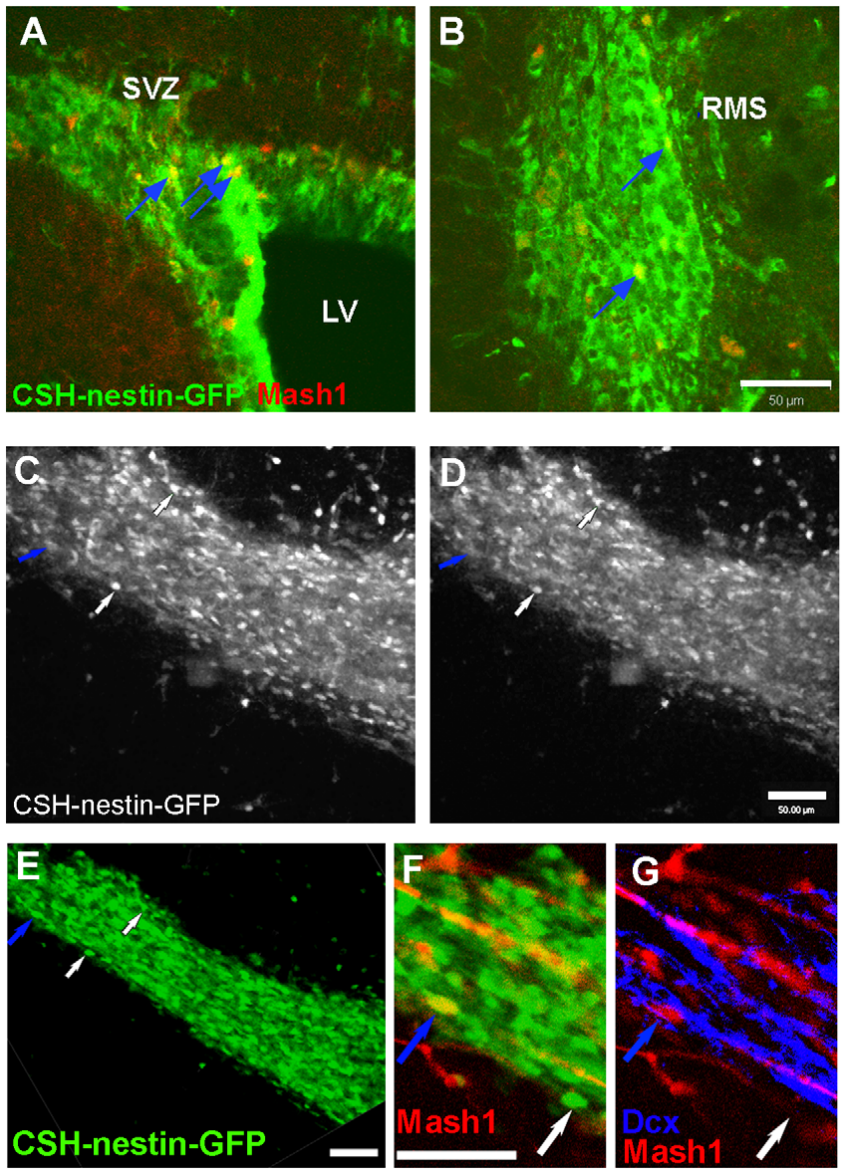
Mash1+ progenitor cells are not motile in the RMS. A–B: Many bright cells from CSH-nestin-GFP slices were colocalized with Mash1 immunohistochemistry (blue arrows) in the SVZ (A) and the RMS (B). Scale bar = 50 µm. C–D: Motility of CSH-nestin-GFP positive cells was followed with two photon imaging. Most bright cells were stationary (ex. white arrows). Blue arrow: example of a cell that was followed with *post hoc* immunohistochemistry (E–G). Scale bar = 50 µm. E–G: the two photon imaged area was found with confocal microscopy. Arrows indicate cells matched with the last frame of two photon imaging. Mash1+ (F) and Dcx-negative (G) cell shown with blue arrow. Scale bars = 50 µm.

We tested whether transit-amplifying progenitor cells are motile by using Mash1 and Dcx as positive and negative phenotypic markers, respectively. Mash1 is a nuclear transcription factor allowing clear examination of colocalization with GFP in the CSH-nestin-GFP mouse (Fig. 1A)[14], [30]. We confirmed the previous report that a subset of bright GFP+ cells correspond to transit-amplifying cells by showing that bright GFP+ cells in the CSH-nestin-GFP mice expressed Mash1 (Fig. 3A,B). We next performed two photon imaging (Fig. 3C–D) followed immediately by *post hoc* Mash1 and Dcx double immunohistochemistry (Fig. 3F,G). The last frame of the two photon movie was matched with the confocal image (compare Fig. 3D and E). After Mash1+/GFP+ cells were identified and confirmed as being Dcx-negative (Fig. 3E–G), motility was assessed in the two photon movies. No Mash1+/Dcx-negative/GFP^bright^ cells were motile (N = 4 mice, 13 cells in the SVZ and 22 cells in the RMS).

To further probe the question of possible transit-amplifying cell motility, we examined the Mash1-GFP mouse [25]. Double immunohistochemistry showed not all Mash1-GFP+ cells expressed endogenous Mash1 in the SVZ and RMS, but the large majority of Mash1-GFP+ cells were arranged in chains and expressed Dcx (Fig. 1B, Figure S1A-C). This suggests that, unlike the endogenous gene, the transgene persists in neuroblasts and is not a specific marker of transit-amplifying progenitor cells. Time lapse imaging of Mash1-GFP (N = 1 mouse and 1 slice) revealed migration patterns in the SVZ and RMS (Movie S2) that were similar to Dcx-GFP+ cell motility [7]. Chains of cells remained stable, individual cells moved in chains, but were difficult to discern because of the high cell density. These results precluded use of this Mash-1-GFP line for analysis of transit-amplifying progenitor cell motility.

In the preceding studies we imaged a small volume of tissue (6.3×10^−3^ mm^3^) for a few hours, and although we sampled a large number of cells, this was still a small percentage of the whole SVZ population. We therefore took another approach and labeled a large population of Mash1+ progenitors *in vivo* using the dye, Cell Tracker Orange (CTO). The dye was injected into the lateral ventricle of Dcx-GFP mice and migration from the SVZ to the RMS checked three days later (Figure S2A,B). Dcx-GFP mice faithfully represent endogenous Dcx expression, labeling all, and only neuroblasts in the SVZ and RMS (Fig. 1B) [7]. Since CTO diffused through the wall of the LV, (Figure S2A1-2), it labeled migratory cells irrespective of their location in the SVZ. In contrast, CTO did not passively diffuse into the RMS (Figure S2B), ensuring that CTO labeled cells in the RMS migrated there from the SVZ. Immunostaining revealed that 97.0±2.7% of CTO+ cells in the RMS were Dcx-GFP+ and Mash1-negative, confirming that the large majority of migratory SVZ cells are Dcx+ neuroblasts (n = 3 mice, total 210 cells counted) (Figure S2C). There were a few CTO+ cells that were Dcx-GFP negative and Mash1 negative, but there were no CTO+ cells that were Dcx-GFP-negative and Mash1+ (transit-amplifying progenitor cells). These results are consistent with the previous result that Mash1+ transit-amplifying progenitor cells do not migrate from the SVZ to the RMS.

### EGFr Expression on SVZ Neuroblasts

**Figure 4:**
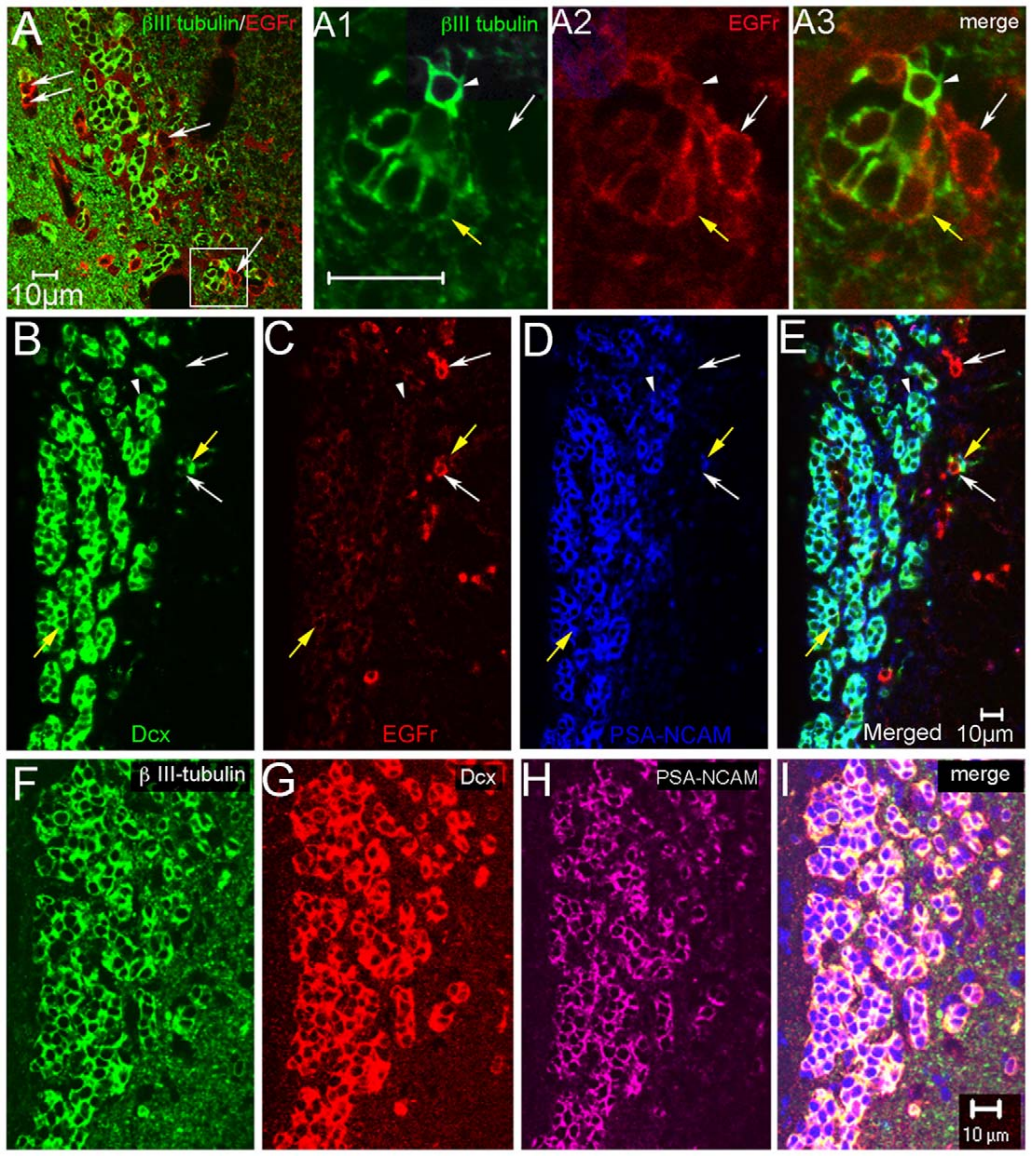
A subset of neuroblasts express EGFr. A: βIII-tubulin and EGFr double immunohistochemistry in a coronal section through the RMS. Many EGFr^high^ cells were βIII-tubulin-negative (white arrows). A1–A3 shows high magnification of inset in A. Note EGFr^high^ cell that is βIII-tubulin-negative (white arrow). Yellow arrow shows a cell that expressed both EGFr and βIII-tubulin. Cells that expressed the highest levels of βIII-tubulin+ (white arrowhead) had EGFr immunofluorescence similar to background levels. Scale bars = 10 µm. (Please see Movie S3). B–E: Dcx (B), EGFr (C), and PSA-NCAM (D) triple immunohistochemistry in the RMS. Simlar colocalization of EGFr with neuroblasts is seen as in A. White arrows point to EGFr^high^ cells that are negative for Dcx and PSA-NCAM. Yellow arrows point to cells that express immunodetectable levels of all three markers. White arrowheads point to neuroblasts that had EGFr immunofluorescence similar to background levels. F–I: Confocal microscopy shows near perfect overlap between βIII-tubulin, Dcx, and PSA-NCAM in RMS neuroblasts (coronal section).

High levels of EGFr expression have been reported on SVZ transit-amplifying progenitor cells and a few stem cells, but not on neuroblasts [15]. While examining progenitor cell motility, during *post hoc* analysis we serendipitously found a subset of neuroblasts that exhibited weak levels of EGFr immmunofluorescence. We examined this in thin sections and found that EGFr expression was inversely correlated with expression levels of βIII-tubulin (Fig. 4A, A1-A3). Whereas the majority of EGFr^high^ cells were at the periphery of the RMS, the majority of EGFr^low^ cells were in longitudinal arrays in the middle of the RMS (Movie S3), which is consistent with Altman's original description of the location of proliferative and migratory cells, respectively [6]. Our data indicated that a subset of βIII-tubulin+ neuroblasts express EGFr, so we tested them for expression of two other SVZ neuroblast markers, Dcx and PSA-NCAM. Both Dcx and PSA-NCAM showed a similar inverse correlation with EGFr immunohistochemistry (Fig. 4B–E). Moreover, triple immunohistochemistry (N = 3 mice) showed near-perfect overlap of the three neuroblast markers (Fig. 4F–I). These data prompted the following studies examining the potential role of EGFr in SVZ neuroblast motility.

### EGFr^low^ Exhibited Slower and More Complex Motility than EGFr-Negative Neuroblasts

**Figure 5:**
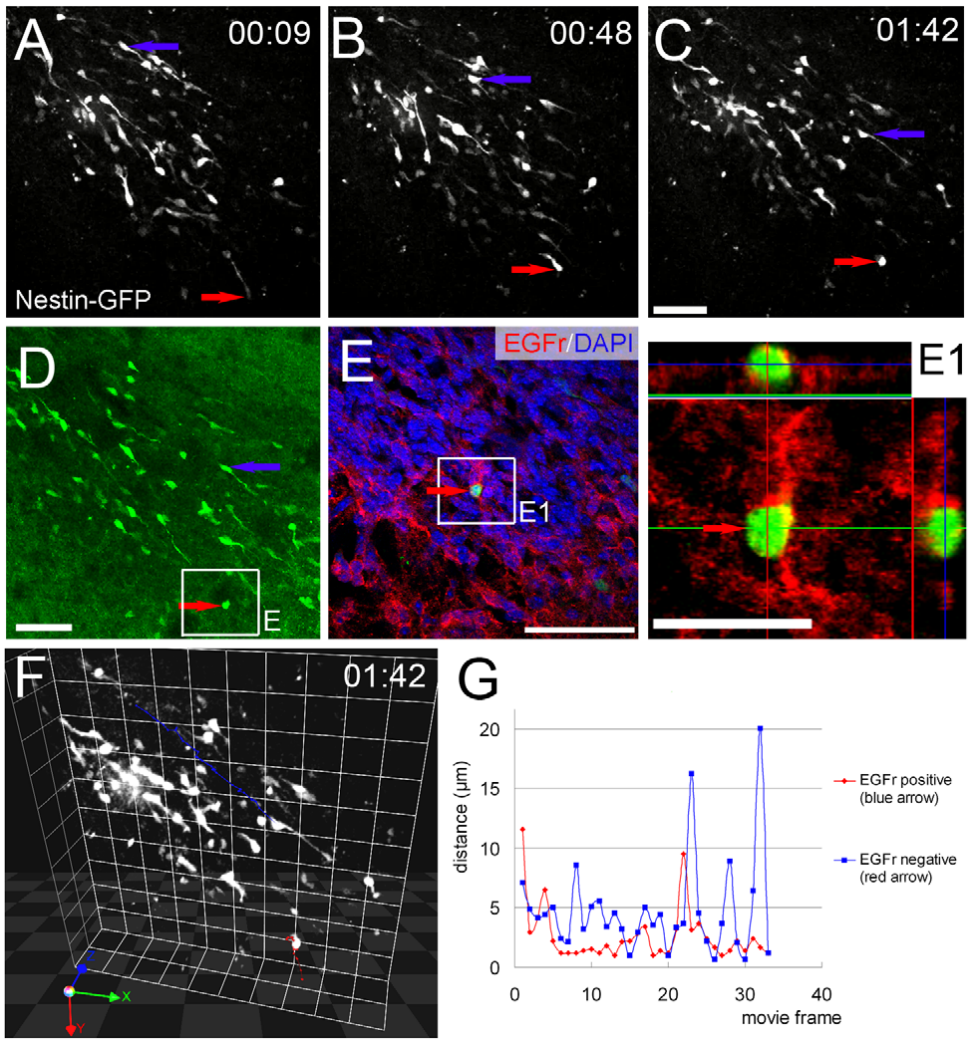
EGFr expression is correlated with differences in motility. A–C: Two photon time lapse imaging of nestin-GFP cells in the RMS. Blue and red arrows indicate migratory and exploratory cells, respectively. Scale bar = 50 µm. (Please see corresponding Movie S4.). D: Confocal image corresponding to two photon image shown in C. Scale bar = 50 µm. E: After fixation and EGFr immunohistochemistry, confocal microscopy was used to find individual cells imaged with two photon microscopy (same cell as in A–C shown with red arrow) Scale bar = 50 µm. E1: high magnification 3-D confocal microscopy showing EGFr^low^ expression (red) on cell exhibiting exploratory motility. Scale bar = 20 µm. F: 3D view of motile cell trajectory. The blue and red trajectories indicate the migratory EGFr negative and exploratory EGFr^low^ cells shown in A–E. 1 unit = 42.9 µm. (Please see corresponding Movie S5.). G: Cell movement distances between frames (3 min apart) of the EGFr-negative (blue) and EGFr^low^ (red) nestin-GFP+ cells shown in A–F. EGFr negative cells were significantly faster than EGFr^low^ exploratory cells.

We first tested if motility patterns or speeds were different in neuroblasts that express EGFr from those that do not. We examined migration using the nestin-GFP mouse line that we previously studied [7], in which a relatively small number of SVZ cells are labeled, allowing individual cell migration analysis (Fig. 1B; Movie S4). Approximately 63% of GFP+ cells in the SVZ are neuroblasts in this line [7]; and consistent with the findings in the present study, we surmised that the only motile GFP+ cells would be neuroblasts. Again, we imaged cells with two photon time lapse microscopy and followed it with *post hoc* immunohistochemistry (Fig. 5A–E, E1). 12.1±1.7% of all nestin-GFP+ cells were EGFr^low^ and most of these, 83.3±16.7%, were motile (N = 3 mice, 93 cells analyzed, average movie length = 1 hr 45 min). As described before [7], cell movement was saltatory (Fig. 5G; Movie S4). EGFr^low^ cells (ex. red arrow and trace in Fig. 5) moved slower (N = 3 mice, 9 cells analyzed) than EGFr negative cells (ex. blue arrow and trace in Fig. 5; 19 cells analyzed) (Table 1, Fig. 5F,G; Movie S4, S5). EGFr^low^ cells also traveled significantly shorter total and net distances than EGFr negative cells (Table 1). In addition to relatively straight rostral movements we recently reported that approximately one third of SVZ cells exhibit local exploratory motility [7]. In the present study we detected both types of movement. The cell in Fig. 5 indicated with the blue arrow exhibited migratory motility, whereas the cell indicated with the red arrow exhibited exploratory motility (Fig. 5A–C, F; Movie S4, S5). The difference in relative straightness of movement is also apparent in 3-dimensional traces (Fig. 5F; Movie S5). The migration index is a simple indication of motility complexity that is independent of speed (MI = net distance/total distance). Nestin-GFP+/EGFr^low^ cells exhibited significantly lower MI (Table 1). These results suggested that EGFr^low^ neuroblasts exhibited slower and more tortuous movements than EGFr-negative neuroblasts.

**Table 1:** Motility comparison between EGFr^low^ cells and EGFr negative cells.

	Total Distance (µm)	Net Distance (µm)	Speed (µm/hr)	Migration index
**EGFr negative cells**	137.0±11.4	89.7±12.4	83.0±6.9	0.60±0.06
**EGFr^low^ cells**	95.3±14.8*	41.9±14.1*	59.6±9.2*	0.37±0.08*
*P<0.05				

### TGF-α Decreased the Percentage of Migratory Neuroblasts

**Figure 6:**
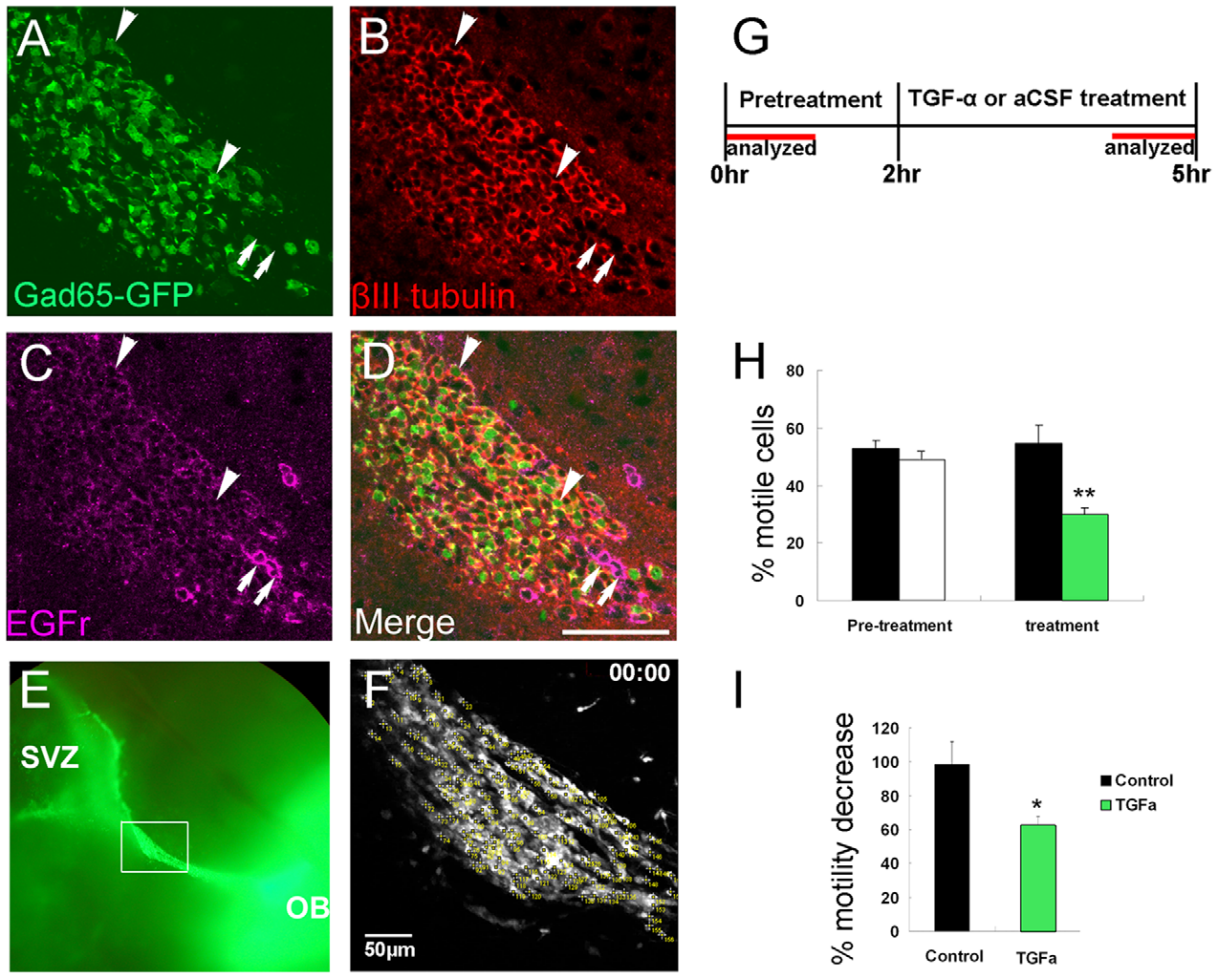
TGF-α decreased the percentage of motile cells. A–D: Gad65-GFP labels a subset of βIII-tubulin+ neuroblasts (arrowheads), but not EGFr^high^ progenitor cells (arrows). E: Low magnification view of Gad65-GFP sagittal section showing area analyzed. F: First frame of two photon time lapse imaging. Each distinguishable cell was labeled (yellow numbers) and analyzed to determine cell motility. Corresponds to first frame of Movie S6 (pretreatment). G: TGF-α schedule and analyzed segments. H: Percentage of motile cells before and after TGF-α treatment. TGF-α caused significant decreases compared to pretreatment and aCSF. **P<0.01. I: Percentage decrease after TGF-α compared to pre-treatment. *P<0.05.

Since EGFr^low^ cells exhibited different patterns of motility than EGFr negative cells, we reasoned that EGFr stimulation may further affect SVZ neuroblast migration. To directly test this, we perfused 10 ng/ml transforming growth factor alpha (TGF-α), an EGFr selective agonist, during two photon time lapse imaging. In this experiment we used a Gad65-GFP mouse in which the majority of SVZ neuroblasts are labeled and cells are still distinguishable from each other (Fig. 1B, 6A–D) [7]. We confirmed that all periglomerular lineages were represented (Figure S3). We imaged the RMS (Fig. 6E) with two photon microscopy for 5 hours: 2 hours (pre-treatment) followed by perfusion with either 10 ng/ml TGF-α or aCSF (control) for 3 hours (Fig. 6G; Movie S6). We analyzed the first hour of pre-treatment and the last hour of TGF-α or aCSF treatment. We previously showed that only a subset of neuroblasts are motile, suggesting that regulating the percentage of motile cells may be a robust mechanism for controlling SVZ neurogenesis [7]. After TGF-α treatment there was a 37.5% loss in the number of motile cells (Fig. 6I). In the pretreatment group 50.9±2.0% of Gad65-GFP cells were motile (total 723 cells traced, n = 8 slices, one slice per mouse) (Fig. 6H). Exposure to TGF-α resulted in only 29.8±2.1% of cells remaining motile (Fig. 6H) (P = 0.008). In contrast, the percent of motile cells in the fifth hour compared to the first hour was 98.5%±12.9 in controls, showing very little loss of motility. This result suggests that stimulation of EGFr decreased the percent of motile cells in the RMS. To examine the direct effect of TGF-α on EGFr^low^ neuroblasts, we performed posthoc EGFr immunohistochemistry on Gad65-GFP slices after TGF-α treatment (Fig. 6G). It was very difficult to identify EGFr^low^ neuroblasts and trace them during the entire 5 hr time-lapse imaging because most expressed GFP at low levels. None of the EGFr^low^/Gad65-GFP cells exhibited fast and unidirectional movement, most were non-motile (81.8%), and even motile EGFr^low^/Gad65-GFP cells moved very slowly in the last hour of imaging (after 2 hr TGF-α treatment) (N = 11 cells, N = 3 mice). The cells we followed through the entire imaging (N = 3 cells) moved slowly in pre-treatment but then stopped during most of the TGF-α treatment. These results suggest a direct effect of EGFr on EGFr^low^ neuroblasts.

We next examined patterns and speeds of motility (N = 40 cells in 4 control slices; N = 39 cells in 4 TGF-α treated slices). The average speed (83.4±2.3 µm/hr for control, 79.5±4.9 µm/hr for TGF-α), motility pattern (exploratory, intermediate, and migratory; 45±11.9%, 22.5±8.5%, and 32.5±14.4% for control, 42.5±19.7%, 12.5±9.5%, and 38.7±19.4% for TGF-α), total distance (86.4±2.8 µm for control and 82.9±6.7 µm for TGF-α), and net distance (43.0±7.2 µm for control and 42.0±8.0 µm for TGF-α) were not significantly different between control slices and the TGF-α treated group. This was not surprising because our results showed that after TGF-α treatment most of the actively migrating cells were EGFr negative neuroblasts.

Finally, we checked whether cell death and proliferation were affected by the TGF-α treatment. This acute 3 hr TGF-α treatment did not significantly change cell death as measured by the number of caspase-3+ cells in the RMS (data not shown). There was a trend towards increased cell proliferation as indicated by the number of PH3+ cells in the RMS (CTL 204.9±24.6 vs. TGF-α 264.5±81.4), but this was not statistically significant.

## Discussion

One of the principle aims of this study was to test the hypothesis that SVZ stem and/or progenitor cells are migratory. Using two photon time lapse microscopy of slices from multiple GFP+ mouse lines we did not find any evidence of motile stem or progenitor cells. Unexpectedly, we found that a marker of transit-amplifying progenitor cells, EGFr, was also expressed by a subset of neuroblasts. Interestingly these EGFr^low^ neuroblasts exhibited slower and more complex motility than EGFr-negative neuroblasts. Moreover, exposure to TGF-α, induced significant decreases in the percentage of migratory neuroblasts. These results do not support the recent evidence of progenitor migration and suggest that EGFr signaling may serve to decrease SVZ neuroblast migration in addition to modulating progenitor proliferation.

### Stem Cells and Transit-Amplifying Progenitor Cells Are Stationary in the SVZ

When ^3^H-thymidine, a mitotic label, was injected and the SVZ examined at short survival times; most forebrain labeled cells were in the SVZ, indicating a large proliferative population [6]. At intermediate time points, labeled cells were found in the RMS, and at long survival times, they had moved to the OB [6]. These classic labeling studies, as well as histological observations, prompted the classification of SVZ cells into distinct proliferative and migratory subpopulations. This model was commensurate with most migratory cells in the developing telencephalon being postmitotic; stem and progenitor cells reside in germinal zones and upon exiting the cell cycle their progeny migrate to final locations. However there are notable exceptions and other clues pointing to the possibility of stem or progenitor cell migration. Neural crest stem cells delaminate, migrate extensively, and continue to divide to generate progeny in the periphery [31]. In the embryonic cortex, intermediate progenitor cells (transit-amplifying cells) migrate from the ventricular zone to the subventricular zone [32], [33]. Finally, astrocytes can migrate [34], [35] and the SVZ is a pro-migratory environment. Taken together, this suggested that GFAP+ astrocyte-like stem cells or transit-amplifying progenitor cells of the adult SVZ may be capable of motility. This is important, because if stem or progenitor cells are motile in the SVZ, they may be able to emigrate and better effectuate repair of injured tissues than the less plastic neuroblasts.

Intriguingly, three separate lines of evidence suggested SVZ stem and/or progenitor cells may be motile. A population of NG2+ cells were identified in the SVZ as transit-amplifying progenitors that migrated to the OB and the hippocampus [8], [9]. However, NG2-Cre lineage tracing experiments indicate that NG2+ cells in the SVZ do not give rise to OB neurons [36]. Instead NG2+ cells in the SVZ are likely to be oligodendrocyte progenitor cells which is consistent with them overlapping with CNPase-GFP [8], [36]. Oligodendrocyte progenitors occupy a distinct caudal subdomain in the SVZ [37], and we did not test for oligodendrocyte progenitor motility in this study. The second line of evidence is from two-photon time lapse microscopy studies showing that a subset of locally motile nestin-GFP+ cells are Dcx-negative [7]. One interpretation of these results is that in addition to neuroblasts, transit-amplifying progenitor cells or GFAP+ cells may be motile [7]. Finally, dopamine depletion combined with TGF-a infusion induces migration of SVZ transit-amplifying progenitor cells into the striatum [14] and intra-cerebroventricular infusion of EGF enhances progenitor cell proliferation and invasion to the striatum [38].

We found no evidence of SVZ stem or progenitor cell motility despite extensively examining the SVZ and RMS, using several lines of GFP+ mice, and searching for both local and long-distance motility. Two lines of mice which label GFAP+ astrocytes and stem cells in the SVZ indicated complete lack of motility. We confirmed that many CSH-Nestin-GFP^bright^ cells were GFAP+ or Mash1+, which was consistent with the antigenic phenotype of SVZ stem and progenitor cells. The CSH-Nestin-GFP mouse dim neuroblasts [27] migrated, serving as an internal control for the stationary bright GFP+ SVZ stem and progenitor cells. Importantly we also employed *post hoc* immunolabeling to confirm the progenitor phenotypes of non-motile cells. Time lapse imaging of mGFAP-GFP SVZ cells also did not show any motility [39]. Astrocytes can be migratory in other contexts, therefore it is not clear why they should not move in the SVZ, which provides an environment favorable to migration of endogenous neuroblasts as well as transplanted cells. Similar to short migration (2 photon experiments), we did not find any evidence of long-distance migration of progenitor cells labeled with CTO.

Our findings are consistent with both Altman's descriptions of the SVZ and the RMS [6], as well as with more recent data showing two largely separate proliferative and migratory populations in the SVZ [4], [40]. It is consistent with data showing that label-retaining cells (stem cells) reside in the SVZ several days after ^3^H-thymidine labeling [4]. Similarly, adenoviral labeled radial glia in the postnatal SVZ gave rise to sessile SVZ astrocytic stem cells [41]. It is still possible that stem and/or progenitor cell motility exists, but that it is just very rare and that more sampling would uncvover it, however we imaged and analyzed 874 cells in our search. Alternatively, their motility may be inhibited by our imaging technique - *in vivo* imaging will ultimately be required to confirm our results. Finally, we can not rule out the possibility that stem or progenitor cells are sessile constitutively but brain injury induces motility.

### EGFr^low^ Neuroblasts Showed Slower Motility

EGFr is one of the most commonly used markers for SVZ progenitors and is not thought to be expressed by SVZ neuroblasts [15]. However this study showed low levels of EGFr expression on some neuroblasts. Similarly, it was recently demonstrated that a subset of CD24^low^ FACsorted mouse neuroblasts express undetectable to low levels of EGFr [3], and that rat neuroblasts express EGFr [42]. We do not know how much of the low EGFr expression detected on SVZ neuroblasts is inherited from their EGFr^high^ precursor cells or transcribed directly, though Cheng and colleagues found EGFr transcripts in SVZ neuroblasts [43]. We noted an inverse correlation between EGFr and βIII-tubulin expression, suggesting that EGFr expression is gradually lost as neuroblasts mature. Alternatively, neuroblast heterogeneity might explain why only a subset express EGFr. However, the three most commonly used markers for SVZ neuroblasts, PSA-NCAM, βIII-tubulin and Dcx were remarkably co-expressed, arguing against heterogeneity.

Several phenotypic, morphological, and functional studies suggest that the transition from GFAP+ stem cells to intermediate transit-amplifying progenitors to migratory neuroblasts is gradual. The majority of EGFr+ cells in the SVZ are thought to be transit-amplifying progenitor cells, which reportedly have simple round morphologies [15]. Yet, we showed that EGFr^high^ cell morphology ranges from round, to bipolar, to multipolar. Similarly, the morphology of motile cells is on a continuum: cells with the classic bipolar migratory morphology, multipolar cells, and round cells [7]. SVZ cells can also switch to behavioral repertoires associated with their progenitors. Despite the evidence for distinct proliferative and migratory populations described above, there is evidence that migrating neuroblasts occasionally proliferate [44], [45]. Remarkably, magnetic bead sorted PSA-NCAM+ SVZ neuroblasts become gliogenic when transplanted, suggesting they lose their fate commitment and acquire stem cell-like characteristics [46]. Transit-amplifying progenitors cells can revert to stem cell-like behaviors when exposed to high levels of EGF [15]. Taken together, we believe these data are compatible with a model of gradual and reversible transitions in the lineage between SVZ stem cells to neuroblasts. Our data that SVZ neuroblasts express EGFr supports this notion.

Our findings prompted the question of what function, if any, EGFr has on SVZ neuroblast migration. EGF is a pleiotropic molecule and EGFr signaling mediates a multiplicity of events in different contexts. Different levels of EGFr selectively drive proliferation and astrogliogenesis during early stages of cerebral cortex development [47]. EGF signaling also regulates adult SVZ cell division *in vivo* and is necessary for *in vitro* generation of neurospheres [18], [19], [48]. Interestingly we showed that among many factors tested, EGF was uniquely increased in the region of cerebral cortex injury to which SVZ neuroblasts migrate [49]. EGFr also drives SVZ derived oligodendrocyte genesis and repair after demyelination [50], [51]. The combined data suggest that EGF and EGFr mediate several different normal and reparative functions of the SVZ. EGF and EGFr signaling mediates migration across several phyla and a wide variety of cells [52]–[55]. They are involved in dorsally directed migration of border cells in invertebrates [56], [57]. In the mammalian telencephalon, asymmetric cell division results in daughter cells expressing different levels of EGFr [22]. EGFr overexpression induced motility in non-motile telencephalic cells [9], [20]. EGFr^high^ cells migrate up EGF gradients in the cerebral cortex and lateral migratory stream but not in the RMS, suggesting that SVZ/RMS neuroblasts respond differently to EGFr signalling [21].

In this study we found motile cells that expressed EGFr. We believe that the nestin-GFP+/EGFr^low^ cells are neuroblasts since EGFr^low^ cells expressed βIII-tubulin, Dcx and PSA-NCAM. These results show directly that EGFr expression is not incompatible with migration and expands the behavioral repertoire of EGFr+ cells in the SVZ. Recently, local exploratory SVZ cell motility was seen in nestin-GFP and Gad-GFP mice [7]. Since the former primarily, and the latter exclusively labels neuroblasts, it suggested that neuroblasts can exhibit exploratory movement. Here we found that EGFr^low^ cells are more exploratory and slower than motile cells which do not express EGFr. Moreover, EGFr^low^ cells traveled shorter net and total distances compared to EGFr negative cells. In our previous study, Gad-GFP+ cells, which are thought to be older neuroblasts [58], were significantly faster than nestin-GFP+ cells [7]. Taken together, EGFr^low^ cells showed motility patterns between stationary and migratory cells. One possible explanation is that EGFr^low^ cells are recently born neuroblasts that have not acquired a fully migratory phenotype. Alternatively, EGFr may be re-expressed when cells slow down or stop.

### Pharmacological EGFr Stimulation Decreased Percentage of Motile Cells

Previously, we showed that only a subset of SVZ neuroblasts are migratory at any given time [7], and here we demonstrate that exposure to the EGFr agonist TGF-α significantly decreased the percentage of migrating cells. Changing the ratio of migratory to non-migratory cells would greatly impact newborn neuron migration rates to the OB. Our results are compatible with data showing that EGF infusion decreases the number of newborn neurons that have migrated to the OB [48]. One potential explanation is that TGF-α acted directly on EGFr^low^ neuroblasts. To support this argument, we observed EGFr^low^ cells which were motile before TGF-α treatment but stopped after the treatment. The decreased percent of migrating cells in this study could also be explained by increased proliferation; cells theoretically have to stop moving before they divide. Indeed, there was a trend towards increased proliferation, even after the acute 3 hour TGF-α exposure. The short time course also suggests that TGF-α acted directly on EGFr^low^ neuroblasts, although we can not rule out an indirect effect via stimulation of EGFr^high^ cells and release of soluble factors from them. Regardless of whether either scenario is correct, TGF-α treatment decreased the percentage of motile cells. Several studies have shown that EGF or TGF-α infusion induce SVZ neuroblast emigration to the striatum [14]–[17], [23], [24]. Although we did not observe cell emigration to the striatum in slices after TGF-α, it may be that longer stimulation is needed for this effect. TGF-α treatment did not change speed, migration distance, and motility pattern of migrating cells.

Taken together, the data suggest that EGFr stimulation may first slow down and then inhibit normal migration in the SVZ and RMS, and in pathological contexts stimulate its emigration to adjacent nuclei. It also suggests that the high levels of EGFr on stem and progenitor cells may inhibit this motility. The dynamics between proliferation and migration are important to coordinate for regulating neurogenesis. Another member of the EGFr family, ErbB4, is necessary for normal migration during brain development [59]. SVZ neuroblasts express ErbB4, infusion of its ligand neuregulin-1 induces and maintains motility, and the RMS of ErbB4 conditional nulls is severely disrupted [60], [61]. Instead of promoting migration, our data suggests stimulation of EGFr, also known as ErbB1, decreases migration. Thus ErbB1 and ErbB4 signaling may serve to balance each others' effects on SVZ proliferation and migration.

### Conclusion

In conclusion, our data confirm early reports of a clear distinction between proliferative and migratory behaviors and argue against stem and progenitor migration. They also indicate that one possible mechanism balancing them is EGFr signaling.

## Supporting Information

Figure S1 Mash1-GFP labels Mash1 progenitor cells and neuroblasts. A–B: Mash1 immunohistochemistry on Mash1-GFP mice in the SVZ (A) and the RMS (B). Note that some immunolabeled Mash1+ cells were Mash1-GFP+ (arrows) whereas other Mash1+ cells were not Mash1-GFP+ (arrowheads). C: Dcx immunohistochemistry in the RMS showed that most Mash1-GFP+ cells were colocalized with Dcx in the RMS (arrows).
(2.58 MB TIF)

Figure S2 Only neuroblasts migrate from the SVZ to the RMS. A: Schematic of CTO injection, A1: shows injection track into lateral ventricle, A2: shows diffusion of CTO to the contralateral lateral ventricle. B: Sagittal section showing CTO injection (red) in the LV of a Dcx-GFP+ mouse. C: Coronal section, the majority of CTO+ cells (red) were also Dcx-GFP+ (green, arrows) but were not Mash1+ (blue, arrowhead). C1. Confocal orthogonal view of the cell shown with left arrow.
(8.76 MB TIF)

Figure S3 Gad65-GFP+ cells give rise to all three major periglomerular cell layer subtypes. Recent studies have shown that different parts of the SVZ generate different types of interneurons in the OB [41], [62]. We tested if Gad65-GFP+ cells belong to specific sublineages of OB cells, and found GFP+ cells that were tyrosine hydroxylase+ (TH)(A), calretinin+ (CalR)(B), or calbindin+ (CalB)(C) consistent with a previous report [28]. These results showed that we were not studying selective sublineages of SVZ neuroblasts. Arrows show examples of co-labeled cells. Scale bar = 50 µm.
(2.77 MB TIF)

Movie S1 Movie from a CSH-nestin-GFP slice in the RMS, note that bright GFP+ cells are stationary. Playing movie at high speeds allows optimal visualization of dim cell movement. Corresponds to Fig. 2E–F. Frame is 353×353×51 (x, y, z) µm.
(1.57 MB MOV)

Movie S2 Movie from a Mash1-GFP+ slice at the elbow of the RMS. Note the visible chain migration because of GFP retention in migrating neuroblasts. Cells appear to be moving in multiple different directions in the ventral portion.
(2.66 MB MOV)

Movie S3 EGFr immunohistochemistry in the vertical limb of the RMS, DAPI counterstain. Confocal stack of 32 optical sections, each separated by 0.37 microns. Note the preponderance of EGFrhigh cells at the edge of the RMS, many of which appear to be clustered. Within the RMS, a range of high to weak EGFr immunofluorescence is detected in a pattern suggesting expression by chain migrating neuroblasts. EGFr+ cells had a continuum of morphologies ranging from bipolar to round.
(0.49 MB MOV)

Movie S4 Movie from a Nestin-GFP slice in the RMS. Note individual cells move with different migratory patterns. Slice was fixed and immunostained for EGFr, shown in Fig. 5. Frame is 284×295×51 (x, y, z) µm.
(0.72 MB MOV)

Movie S5 3D tracing of Movie 4. Blue and red trajectories correspond to migratory and exploratory cells shown with blue and red arrows in Fig. 5 and Movie 4. Note the difference between migratory (blue) and exploratory (red) motility patterns.
(1.03 MB MOV)

Movie S6 Two photon time lapse imaging of a Gad65-GFP slice before (pre-treatment) and during 10 ng/ml TGF-α treatment. The first hour of pretreatment and the last hour of TGF-α treatment is shown and were analyzed. One quarter of the field imaged and analyzed is shown for greater clarity. (Time stamp reset after pretreatment.)
(0.57 MB MOV)
