# Supplementary figures and images for: Adult Mouse Subventricular Zone Stem and Progenitor Cells Are Sessile and Epidermal Growth Factor Receptor Negatively Regulates Neuroblast Migration
Source: PLoS One. 2009 Dec 2;4(12):e8122. doi: 10.1371/journal.pone.0008122 (PMC2780296; doi:10.1371/journal.pone.0008122)

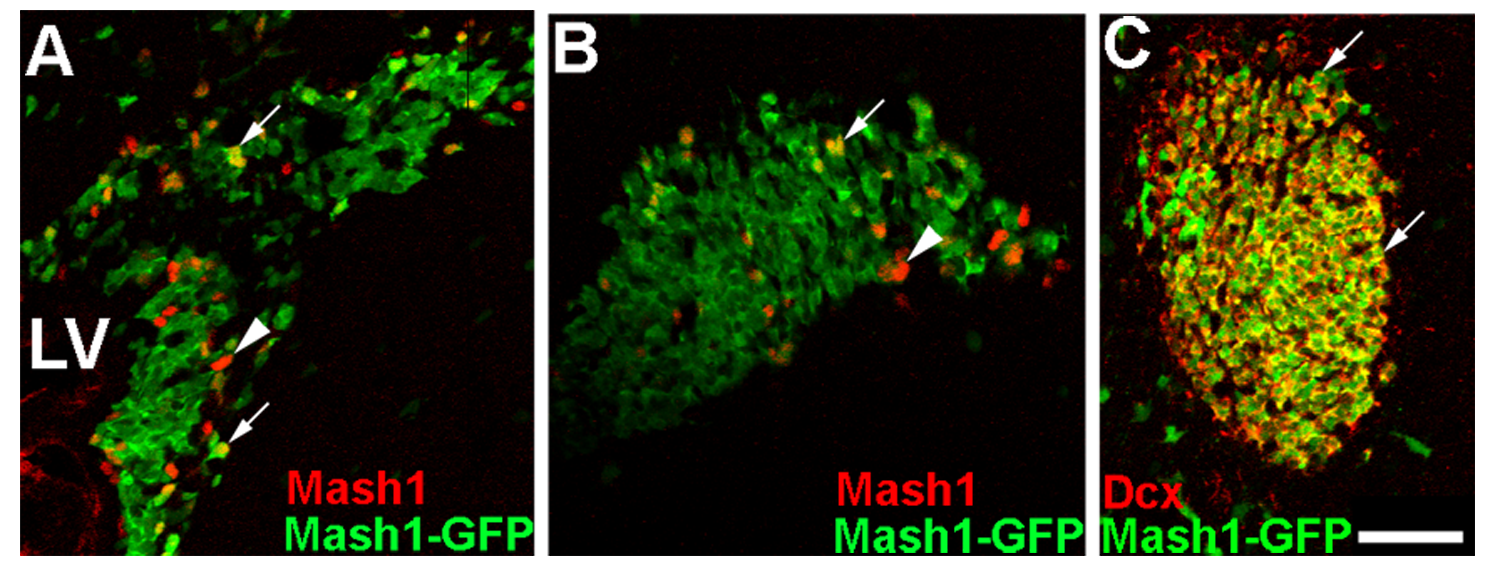

Supplement: Figure S1 — Mash1-GFP labels Mash1 progenitor cells and neuroblasts. A–B: Mash1 immunohistochemistry on Mash1-GFP mice in the SVZ (A) and the RMS (B). Note that some immunolabeled Mash1+ cells were Mash1-GFP+ (arrows) whereas other Mash1+ cells were not Mash1-GFP+ (arrowheads). C: Dcx immunohistochemistry in the RMS showed that most Mash1-GFP+ cells were colocalized with Dcx in the RMS (arrows). (2.58 MB TIF) [file pone.0008122.s001.tif]

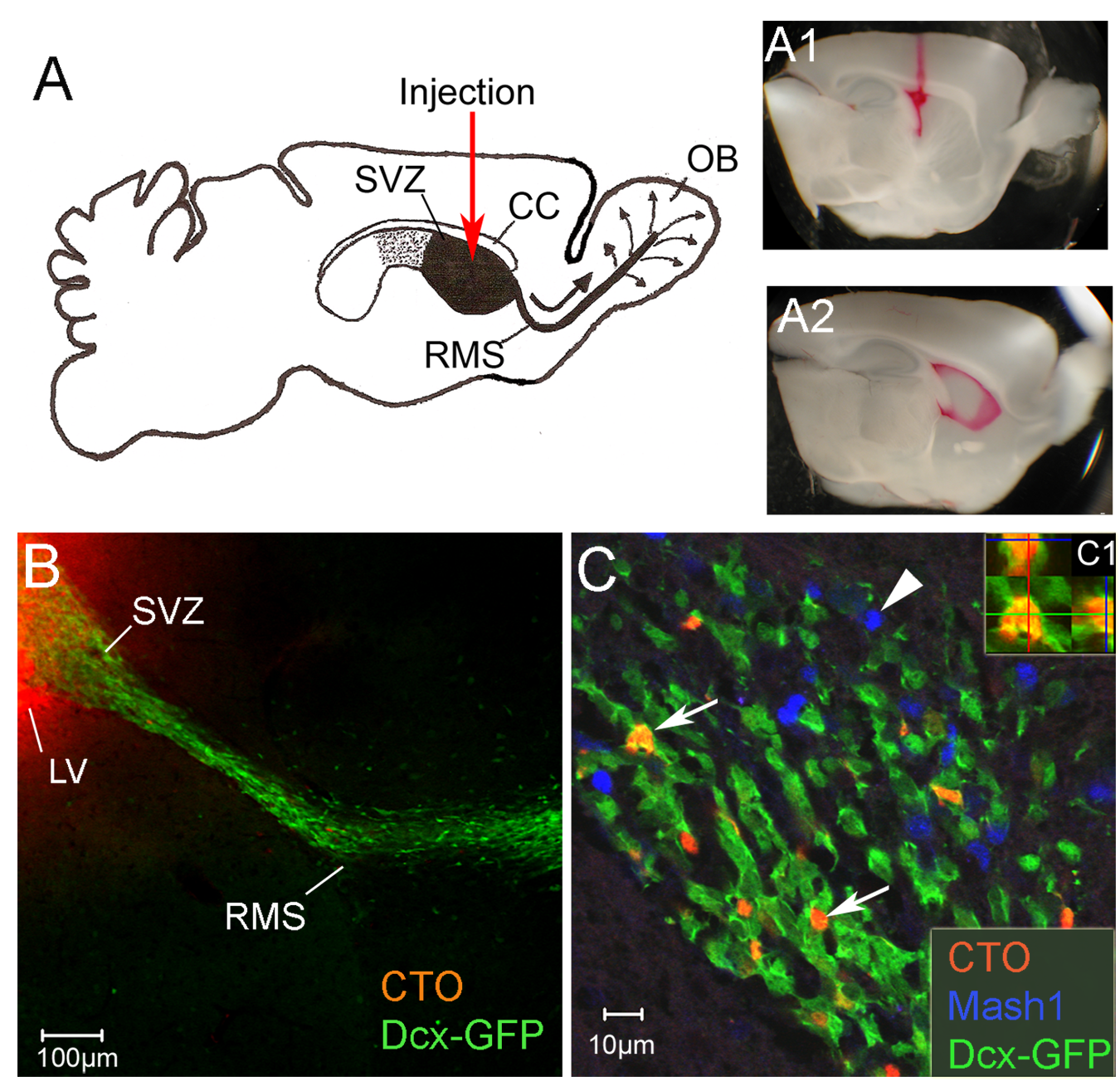

Supplement: Figure S2 — Only neuroblasts migrate from the SVZ to the RMS. A: Schematic of CTO injection, A1: shows injection track into lateral ventricle, A2: shows diffusion of CTO to the contralateral lateral ventricle. B: Sagittal section showing CTO injection (red) in the LV of a Dcx-GFP+ mouse. C: Coronal section, the majority of CTO+ cells (red) were also Dcx-GFP+ (green, arrows) but were not Mash1+ (blue, arrowhead). C1. Confocal orthogonal view of the cell shown with left arrow. (8.76 MB TIF) [file pone.0008122.s002.tif]

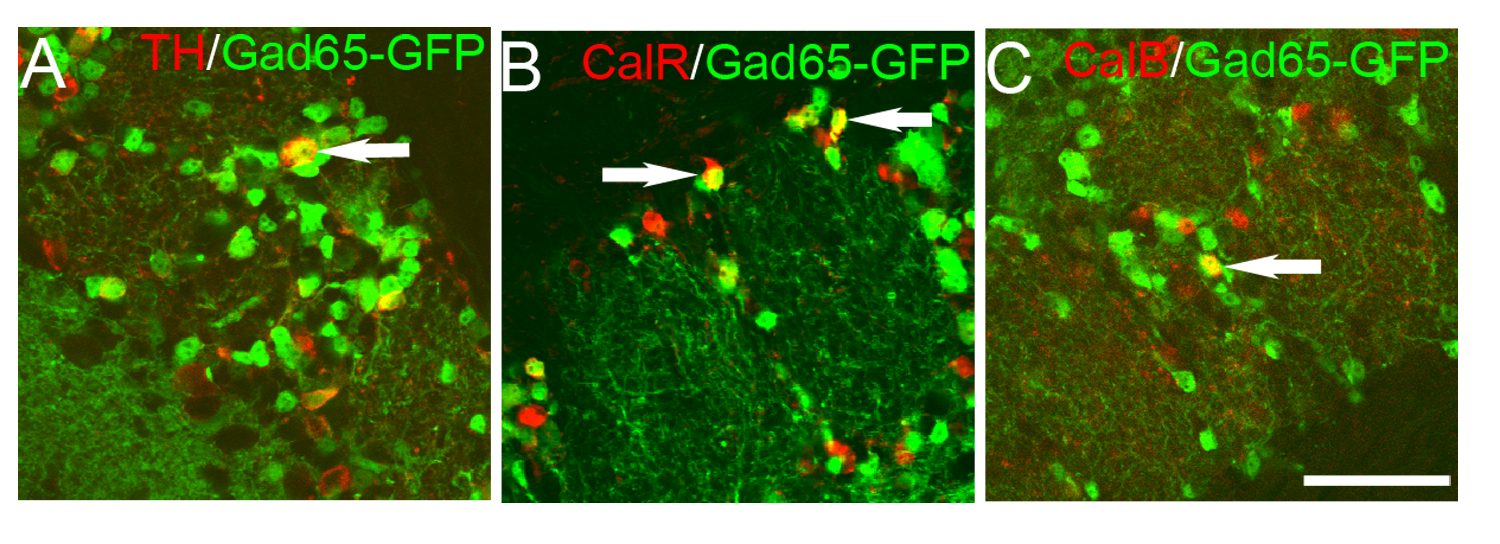

Supplement: Figure S3 — Gad65-GFP+ cells give rise to all three major periglomerular cell layer subtypes. Recent studies have shown that different parts of the SVZ generate different types of interneurons in the OB [41], [62]. We tested if Gad65-GFP+ cells belong to specific sublineages of OB cells, and found GFP+ cells that were tyrosine hydroxylase+ (TH)(A), calretinin+ (CalR)(B), or calbindin+ (CalB)(C) consistent with a previous report [28]. These results showed that we were not studying selective sublineages of SVZ neuroblasts. Arrows show examples of co-labeled cells. Scale bar = 50 µm. (2.77 MB TIF) [file pone.0008122.s003.tif]
